# Supplementary figures and images for: Multifunctional antimicrobial effects of Lactobacillus johnsonii against A/E pathogens Enteropathogenic E. coli and Citrobacter rodentium
Source: Front Immunol. 2026 Jul 10;17:1749001. doi: 10.3389/fimmu.2026.1749001 (PMC13395706; doi:10.3389/fimmu.2026.1749001)

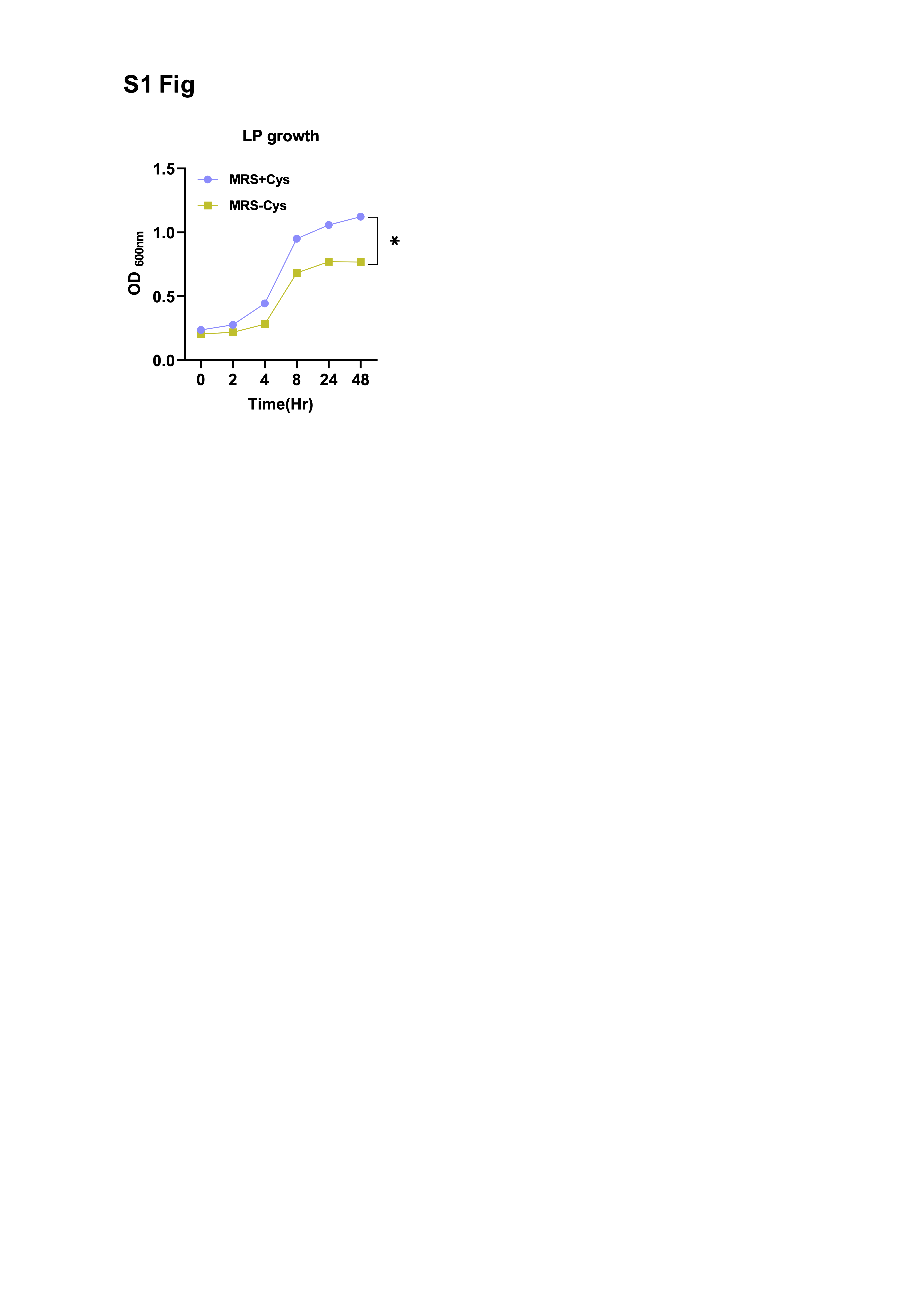

Supplement: Supplementary Figure 1 — Growth curves of L. plantarum in de Man, Rogosa and Sharpe (MRS) broth supplemented with 0.05% (w/v) cysteine (MRS+Cys) versus unsupplemented MRS (MRS-Cys) over 0-72 h incubation at 37 °C. Optical density at 600 nm (OD600) was recorded at the indicated time points. Data are mean ± SEM from three independent biological replicates (n=3). Statistical analysis was performed using one-way ANOVA with Dunnett’s multiple comparisons test; *p< 0.05; ns, not significant. [file Image1.tiff]

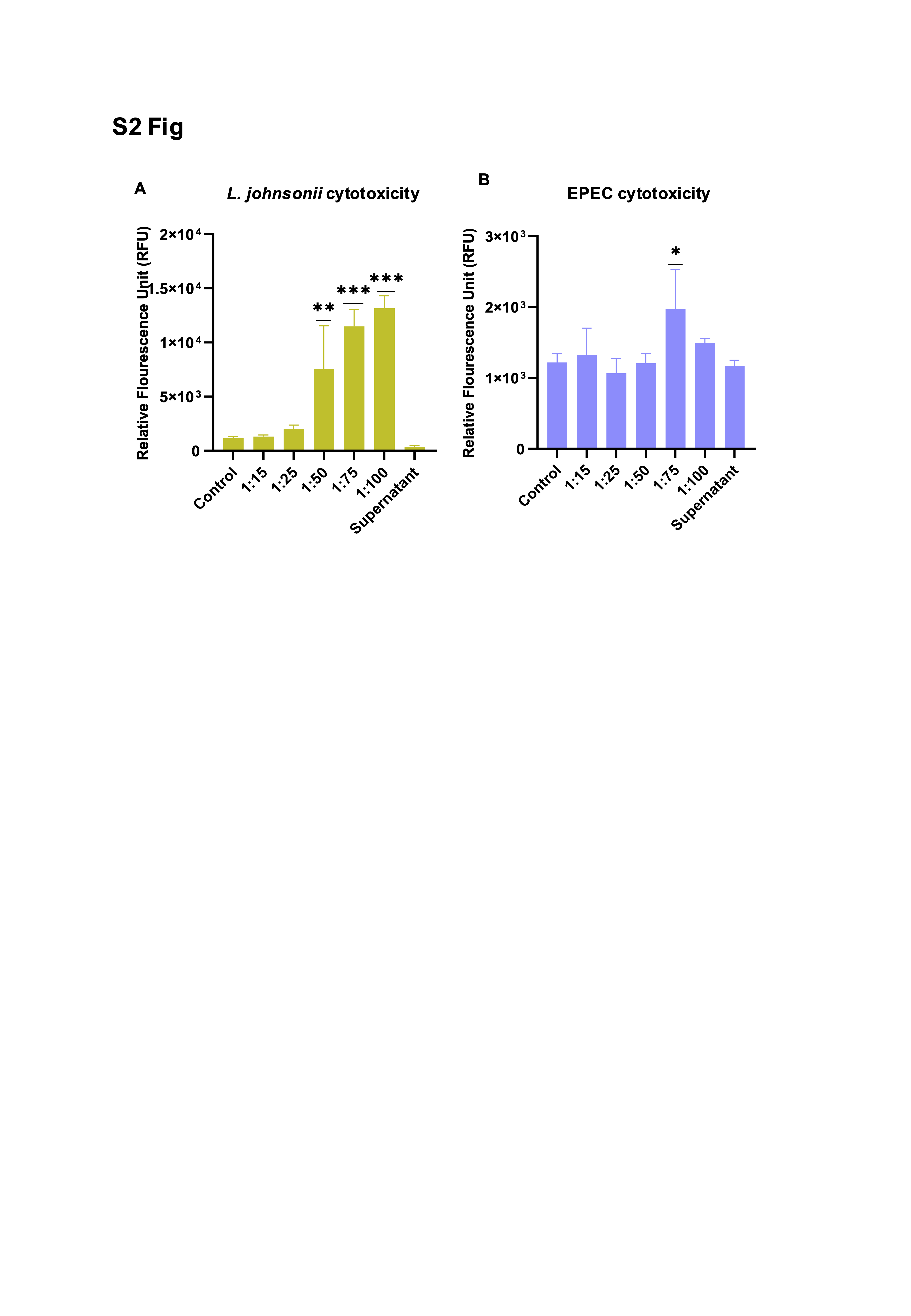

Supplement: Supplementary Figure 2 — Cytotoxicity induced by L. johnsonii and EPEC in HCT-116 cells. (A) Lactate dehydrogenase (LDH) release from HCT-116 intestinal epithelial cells after 6 h exposure to L. johnsonii at different multiplicities of infection (MOI). (B) LDH release from HCT-116 cells after 6 h exposure to EPEC at different MOIs. LDH release (%) was calculated relative to maximum release induced by cell lysis (positive control) and untreated cells (negative control). Data represent the mean ± SEM from two independent experiments performed in triplicate. Statistical analysis: one-way ANOVA *p< 0.05; **p< 0.01; ***p< 0.001. [file Image2.tiff]

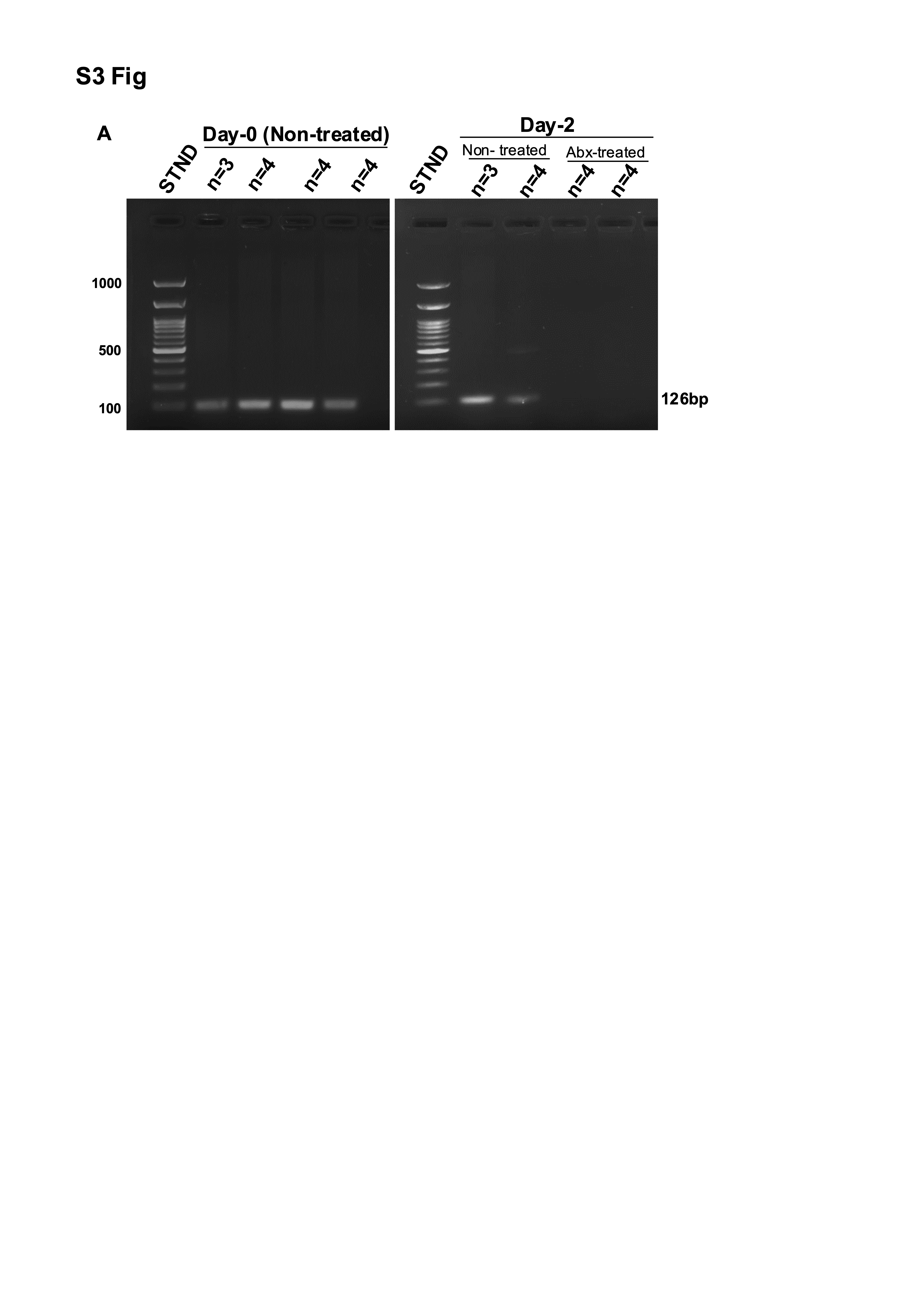

Supplement: Supplementary Figure 3 — Early depletion of L. johnsonii following antibiotic treatment. (A) Agarose gel electrophoresis showing absence of the L. johnsonii-specific 100 bp PCR amplicon in pooled stool DNA from antibiotic-treated mice by Day 2. DNA was extracted from stool samples of n = 4 mice per group and pooled before analysis. Lane 1 and Lane 6: 100 bp DNA ladder; Lanes 2-5: pooled stool DNA samples from Day 0 (pre-antibiotic treatment); Lanes 7-10: pooled stool DNA samples from Day 2 post-antibiotic treatment. [file Image3.tiff]

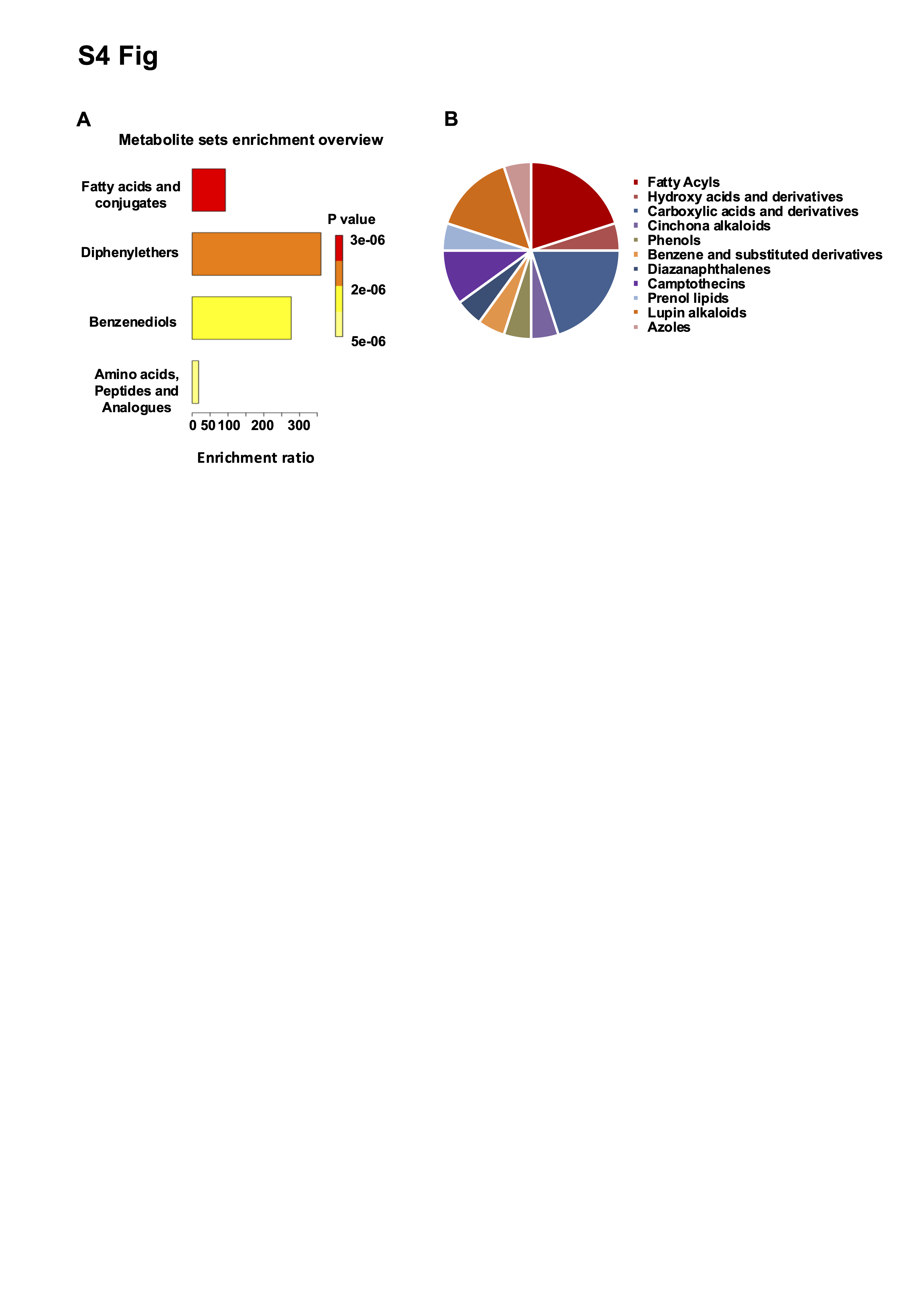

Supplement: Supplementary Figure 4 — Metabolite profiling of bioactive antimicrobial fraction S6 from L. johnsonii.(A) Metabolite set enrichment analysis of the FPLC-purified S6 fraction from L. johnsonii cell-free supernatant, showing significant enrichment of antimicrobial-associated chemical classes, including fatty acyls, hydroxy acid derivatives, carboxylic acids, and diphenylethers. Bars represent enrichment ratio with corresponding p-values. (B) Chemical class distribution of 20 putatively annotated metabolites identified by mass spectrometry in positive and negative ion modes, with predominance of fatty acids, hydroxy acid derivatives, alkaloids, and azoles. Of these, 12 putative metabolites have reported antimicrobial activity, and 4 are specifically active against Escherichia coli (see S4 Table for details). [file Image4.tiff]

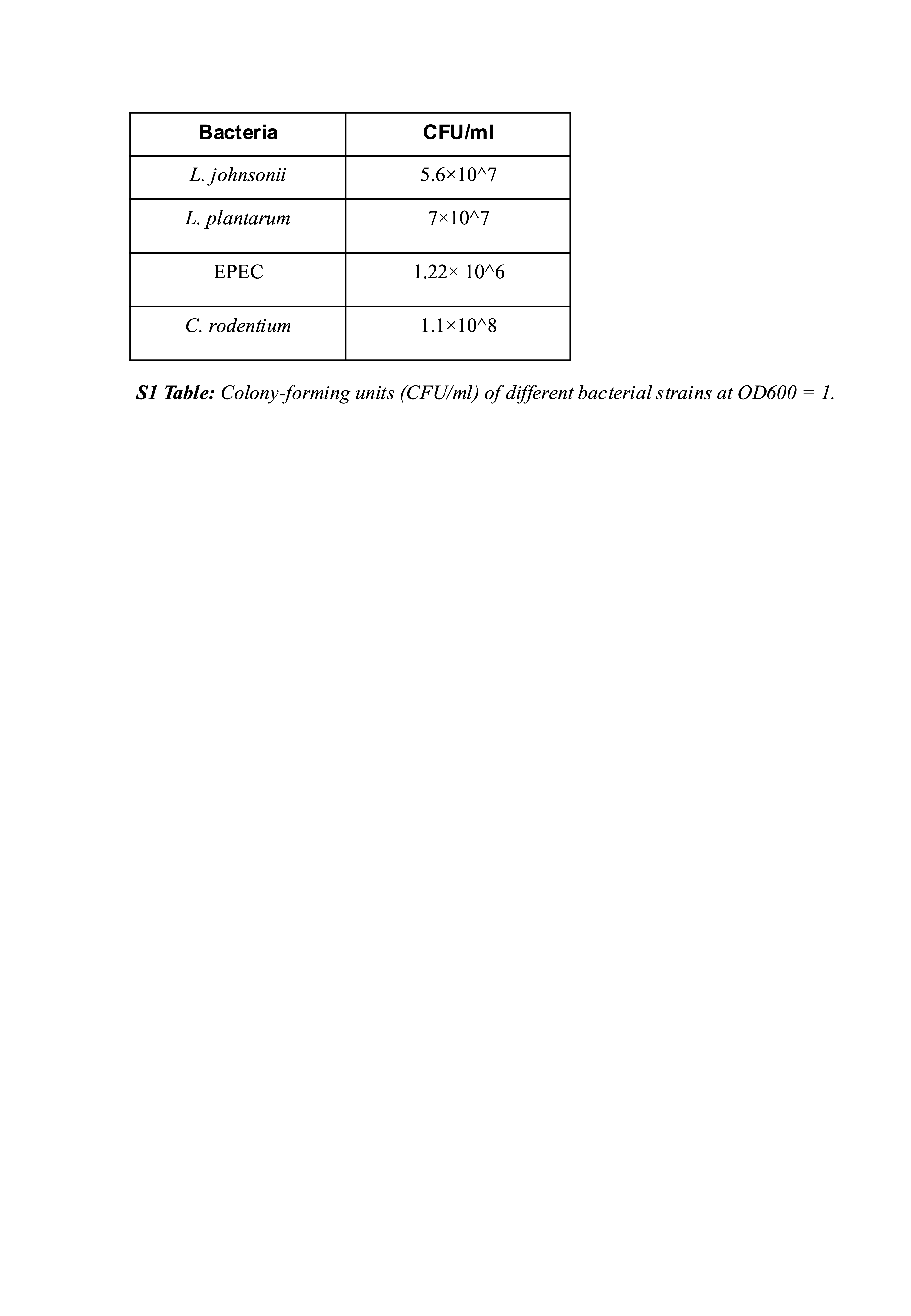

Supplement: Supplementary Table 1 — CFU/mL of different bacterial strains at OD600 = 1. [file Supplementaryfile1.tiff]

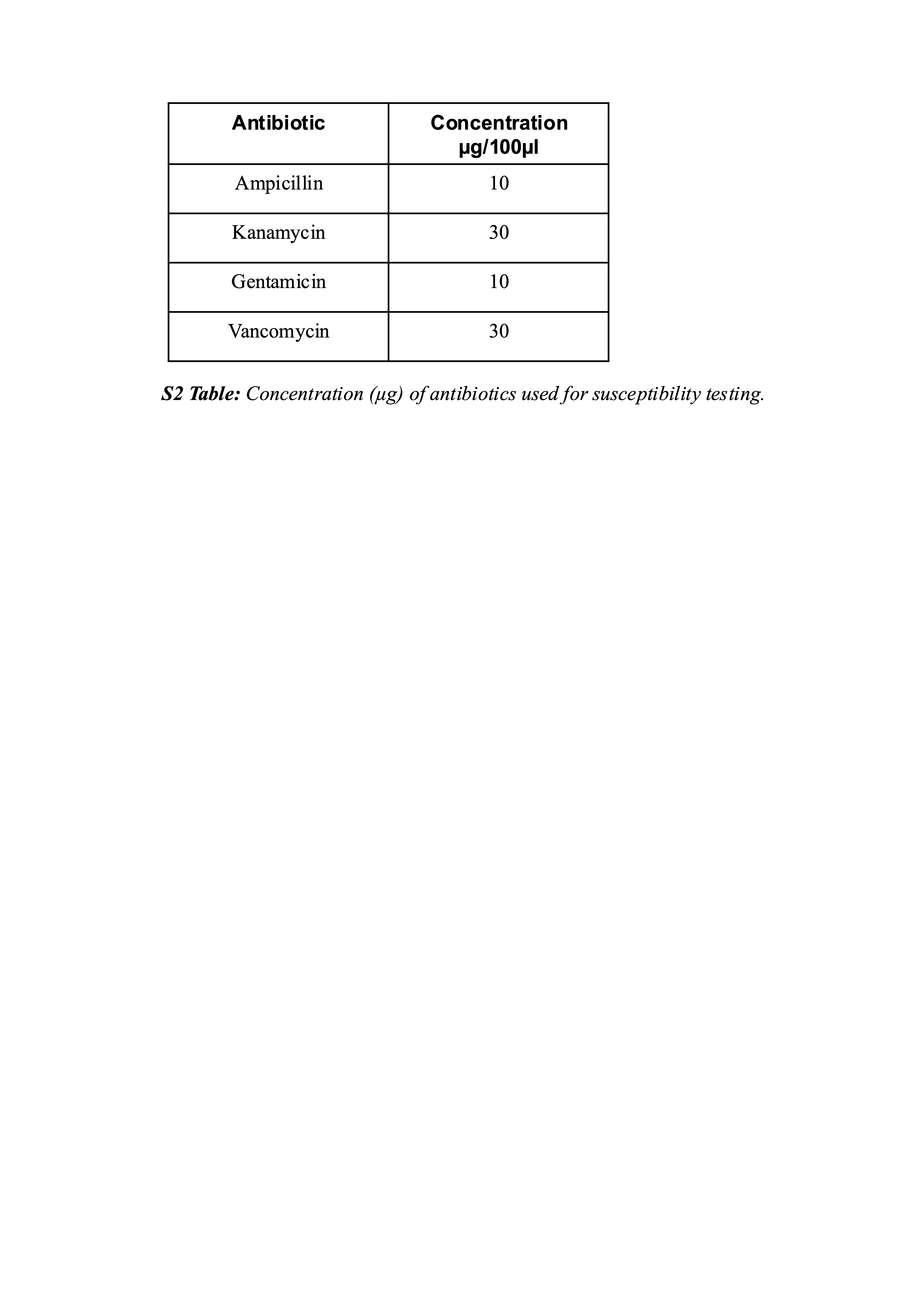

Supplement: Supplementary Table 2 — Concentration (µg) of antibiotics used for probiotic susceptibility testing. [file Supplementaryfile2.tiff]

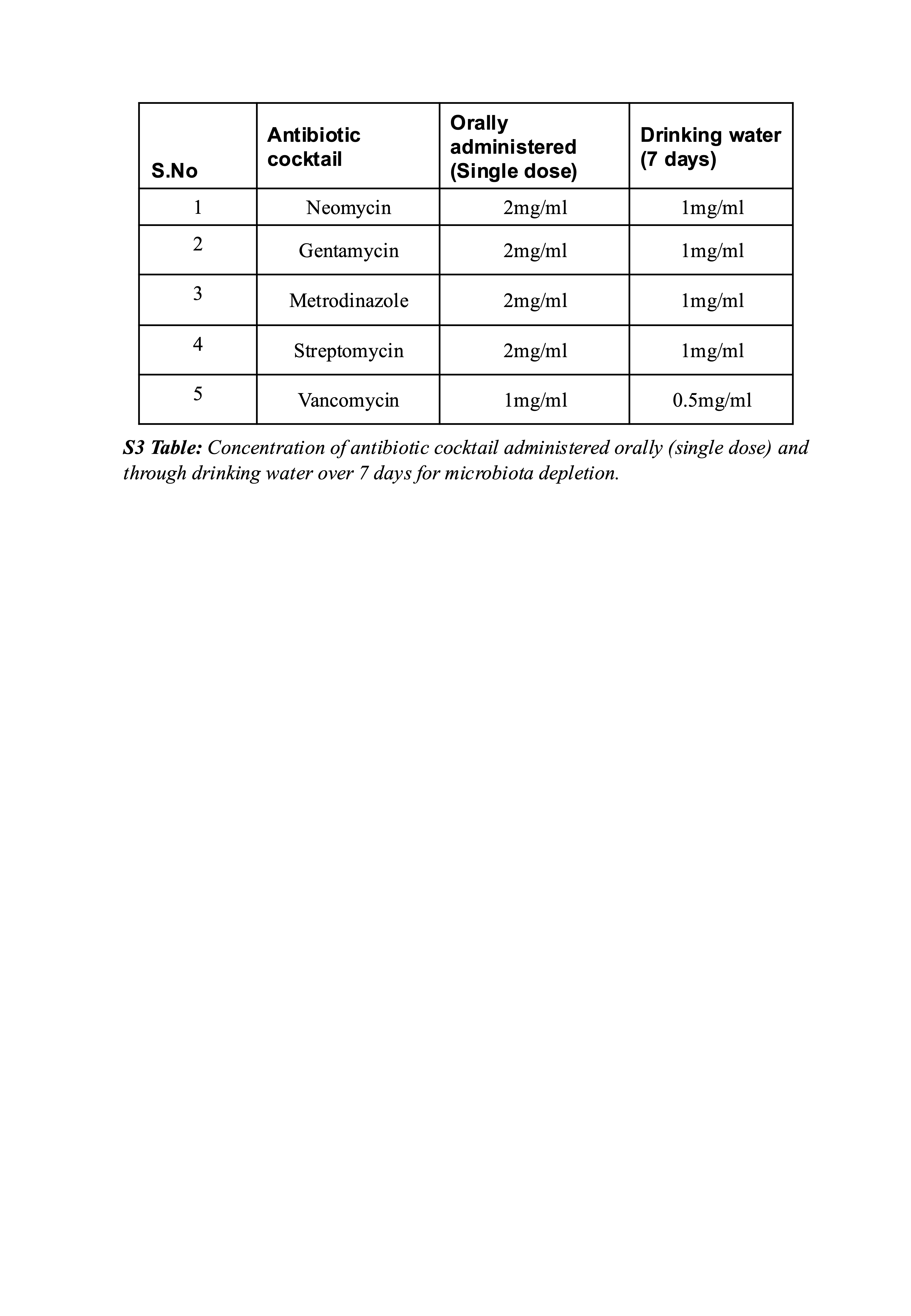

Supplement: Supplementary Table 3 — Concentration of antibiotic cocktail administered orally (single dose) and through drinking water over 7 days for microbiota depletion. [file Supplementaryfile3.tiff]

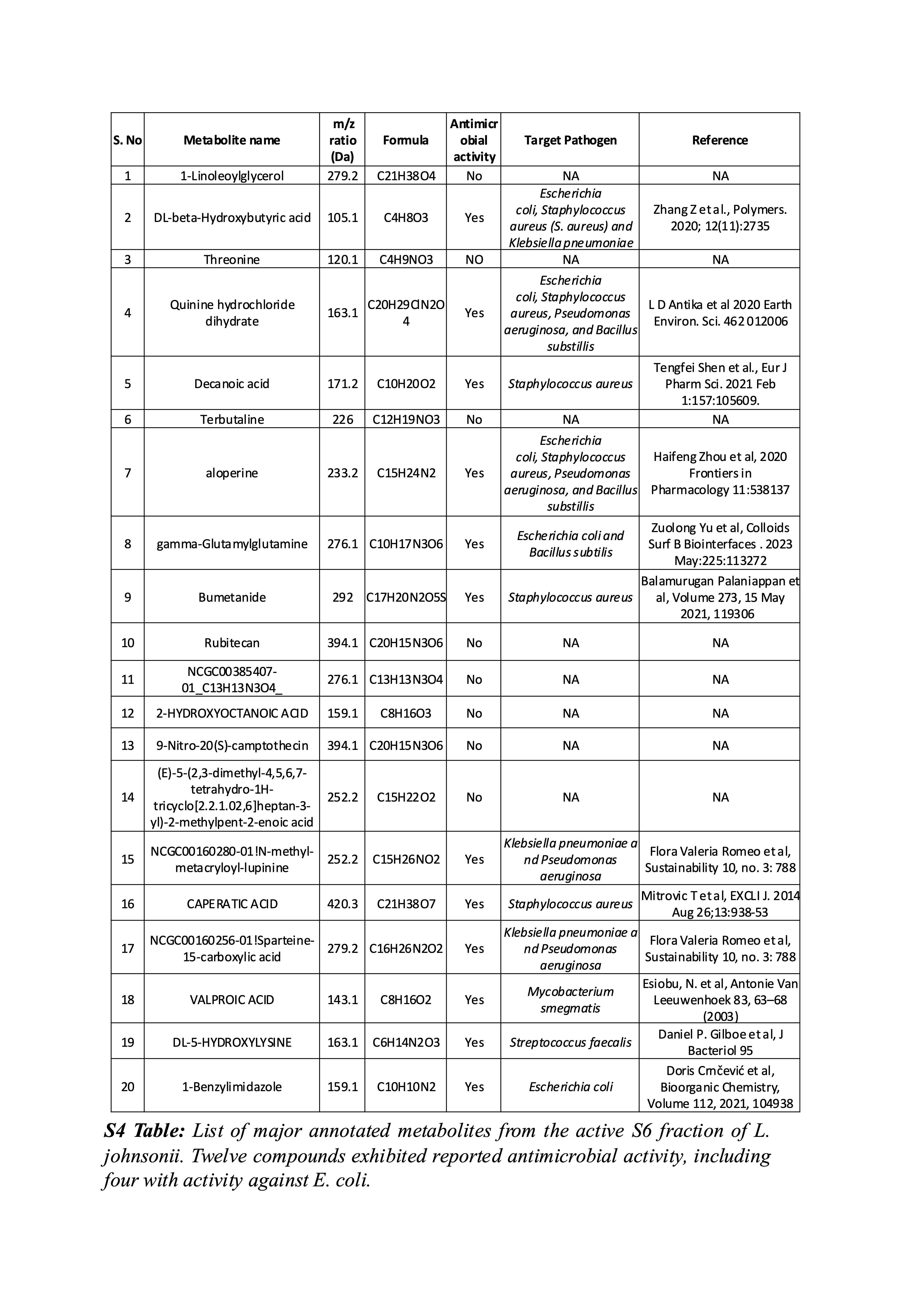

Supplement: Supplementary Table 4 — List of major annotated metabolites from the active S6 fraction of L. johnsonii. Eleven compounds exhibited reported antimicrobial activity, including four with activity against E. coli. [file Supplementaryfile4.tiff]
